# Prevalence, Trends and Associated Risk Factors of Over-weight/Obesity among Rural-to-Urban Migrant Children in China

**Published:** 2019-10

**Authors:** Jinkui LU, Yongsheng XU, Jianming XIANG

**Affiliations:** School of Sport, Shangrao Normal University, Jiangxi Shangrao, 334000, P.R. China

## Dear Editor-in-Chief

Childhood overweight/obesity represent a new worldwide epidemic (1, 2). “The worldwide prevalence of childhood overweight and obesity rate was 4.2% in 1990 and increased to 6.7% in 2010” (2). In 2011, the prevalence of childhood obesity exceeded 20% (3). Similar prevalence was observed in China, underlining a serious public health issue (4). The prevalence of over-weight/obesity was not only in urban children but also in rural-to-urban migrant children, while it mainly took place in economically developed areas such as eastern coastal areas (5). The prevalence of overweight/obesity in rural-to-urban migrant children approached that of Shanghai urban children, far exceeding that of rural children (6). Simultaneously, long duration of living in Shanghai, high socioeconomic factors were the key risk of overweight/obesity among rural-to-urban migrant 7–9-yr-old children (7). Although some studies have reported the prevalence of overweight/obesity and its association with risk factors in rural-to-urban migrant children, there is no study which shows variation trend of prevalence of over-weight/obesity and its association with risk factors by longitudinal study.

The present longitudinal study aimed to examine prevalence of overweight/obesity and its association with risk factors among rural-to-urban migrant children in Shanghai, China.

This was a longitudinal study initiated in 2011. The children aged 7–12 yr old were selected from the Baoshan District, Minhang District, Fengxian District, and Songjiang District of Shanghai using a stratified random sampling method. Three primary schools were selected in each district. The children from grade 1 to 6 were enrolled. The data was collected in Sep every 2-year basis; 2011 (n=8889), 2013 (n=9326), and 2015 (n=7863) are reported in the present study. In order to balance the genders and each age group, 250 boys and 250 girls were randomly selected from each age group. Finally, 3000 children were selected in 2011, 2013, and 2015 (9000 children) for statistical analysis.

Physical measurement and questionnaire investigation was conducted. Height and weight were measured a weight-height meter (AZT-120; Wuxi Weighing Apparatus Co., Wuxi, China). Height was recorded at the nearest 0.1 cm. Weight was recorded at the nearest 0.1 kg. Body mass index (BMI) was calculated as kg/m^2^. The diagnosis of childhood obesity was made according to the Screening and Classification Standards for BMI Overweight and Obesity (Adjusted for age and sex) of Schoolage Children and Teenagers in China (8). EpiData 3.1 (Centers for Disease Control, Atlanta, GA, USA) was used to manage data. SPSS 22.0 (IBM, Armonk, NY, USA) was used for data analysis. Two-sided *P*-values <0.05 were considered statistically significant. Logistic regression analysis was used for multi-factor regression analysis. Variables with *P*<0.05 in univariate analysis were included in the multivariate model, using the backward method.

This study was approved by the Ethics Committee of the School of Physical Education and Health, East China Normal University.

**Fig. 1:**
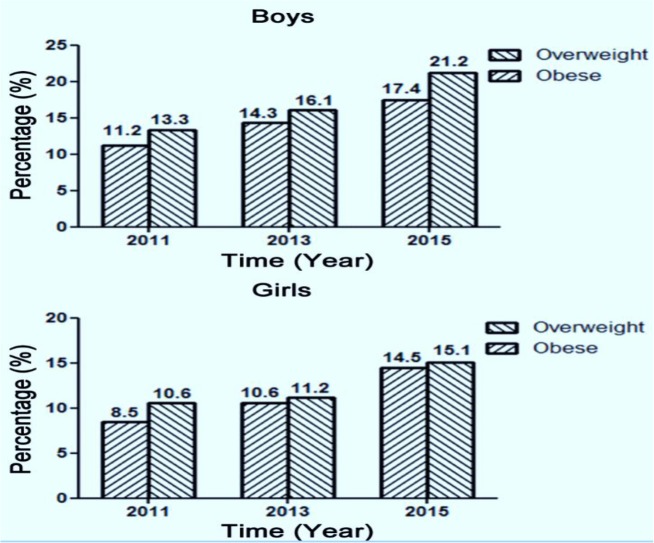
The prevalence of overweight and obesity in three years

Comparison of the basic characteristics of migrant children in Shanghai over the three visits showed that the differences between children with overweight and obesity and between genders were significant (*P*<0.001). The overweight and obesity rate of boys was higher than that of girls, and this rate increased gradually over years (boys vs. girls; 2011: overweight: 13.3% vs. 10.6%, obesity: 11.2% vs. 8.5%; 2013: over-weight: 16.1% vs. 11.2%, obesity: 14.3% vs. 10.6%; 2015: overweight: 21.2% vs. 15.1%, obesity: 17.4% vs. 14.5%; all *P*<0.001) (Fig. 1).

Logistic regression analyses results confirmed that monthly family income, weekly frequency for sports, mother's education level, daily duration of TV/computer, skipping breakfast may affect the status of the weight status of the rural-to-urban migrant children (all *P*<0.05).

The prevalence of overweight/obesity in boys was higher than that of girls, and this prevalence increased gradually over years. The main risk factors changed in time, but mainly included monthly family income, weekly frequency for sports, mother's education level, daily duration of TV/computer, skipping breakfast.
